# Characteristics of human papillomavirus infection among women with cervical cytological abnormalities in the Zhoupu District, Shanghai City, China, 2014–2019

**DOI:** 10.1186/s12985-021-01518-y

**Published:** 2021-03-08

**Authors:** Ping Li, Qing Liu, Wei Li, Zhou Liu, Baoling Xing, Suqin Wu, Zhaoli Zhou, Liping Sun, He Ren, Hengfeng Li, Huaping Li

**Affiliations:** 1 Shanghai University of Medicine and Health Sciences, Shanghai, China; 2 School of Medicine, Renji Hospital, Shanghai Jiao Tong University, Shanghai, China; 3 Changzhou Institute of Technology, Changzhou, China; 4 Shanghai Dunmi Technology Ltd, Shanghai, China; 5 Shanghai University of Medicine and Health Sciences Affiliated Zhoupu Hospital, Shanghai, China

**Keywords:** Epidemiological characteristic, Trend, China, Human papillomavirus, Genotype, ThinPrep cytological test

## Abstract

**Background:**

Human papillomavirus (HPV) infection is currently the main cause of cervical cancer and precancerous lesions in female patients. By analyzing 6-year patient data from Shanghai Zhoupu Hospital in China, we retrospectively analyzed the epidemiological characteristics of women to determine the relationship between HPV genotype and cytological test results.

**Methods:**

From 2014 to 2019, 23,724 cases of cervical shedding were collected from Zhoupu Hospital in Shanghai, China. By comparing the results of HPV and ThinPrep cytology test (TCT), the HPV infection rate of patients was retrospectively analyzed. HPV genotyping using commercial kits can detect 21 HPV subtypes (15 high-risk and 6 low-risk). According to the definition of the Bethesda system, seven types of cervical cytology results were involved.

**Results:**

3816 among 23,724 women, nearly 16.08%, were infected with HPV. The top three highest HPV prevalence rates were high-risk type infection, including HPV52 (3.19%), 58 (2.47%) and 16 (2.34%). The number of single-type HPV infections (3480 (91.20%)) was much larger than the number of multi-type ones (336 (8.8%)). Single-type infections were mainly in women aged 50–60 (16.63%) and women under 30 (15.37%), while multi-type infections were more common in women over 60 (2.67%). By analyzing the long-term trends, between 2014 and 2019, HPV52, 58, and 16 subtypes changed significantly, and the HPV positive rate also changed significantly during this period. Among 4502 TCT positive women, 15 (4.04%), 125 (2.64%),159 (1.54%), 4202 (17.71%) and 1 (0.004%) had atypical glandular cells (AGC), high-grade squamous intraepithelial lesions (HSIL), low-grade squamous intraepithelial lesions (LSIL), atypical squamous cells (ASC)and cervical adenocarcinoma, respectively. The HPV infection rates were 66.08%, 63.99%, 115.20%, 119.50%, and 31.72% for NILM, AGCs, HSILs LSILs and ASCs, respectively.

**Conclusions:**

HPV and TCT screening were very important steps in the secondary prevention of cervical cancer. Through the tracking and analysis of HPV and TCT results in this study, it can provide valuable information for Shanghai's HPV screening and prevention strategies, and provide references for clinical decision-making in the treatment of cervical cancer and precancerous lesions.

## Background

Cervical cancer is the fourth largest cancer prevalence among women in the world More than one-third of cervical cancers worldwide occur in China and India. There are 106,000 cases of cervical cancer and 48,000 deaths in China [1], and the female cancer prevalence and mortality are seventh and ninth respectively [2, 3]. Shanghai is one of the largest cities in China. As the largest one among the 16 districts in Shanghai, the permanent population of Pudong New Area is approximately 5 million (approximately 20% of the total population of Shanghai). From 2002 to 2015, the incidence of cervical cancer in the urban and rural areas of Pudong New Area increased by 9.8 and 15.5% each year. The HPV infection rate and the genotype distribution of Shanghai China have been statistics, not only in the Minhang district [4] but also in the Songjiang for both females [5] and males [6]. Located in the suburbs of Pudong New Area, research on the prevention and screening of cervical cancer in the Zhoupu area is very meaningful [7].

Persistent human papillomavirus (HPV) infection will cause cervical cancer [1] and cervical intraepithelial neoplasia (CIN). According to the degree of affected epithelial tissue, CIN is graded according to grades 1–3, and CIN3 is the most abnormal grade. In this study, CIN1 is equivalent to low-grade squamous intraepithelial neoplasia (LSIL), and ≥ CIN2 is equivalent to precancerous lesions or high-grade squamous intraepithelial neoplasia (HSIL). Patients with high-risk human papillomavirus (HR-HPV) infection usually have multiple sexual partners, which is positively correlated with cervical cancer [8, 9], LSIL, and HSIL infection.

Our previous study confirmed that the HPV infection rate in Zhoupu district was 17.92% (10,670/59,541), of which 86.81% (9263/10,670) was caused by HR-HPV. The six HR-HPV genotypes with the highest positive rate were HPV 52, 16, 58, 53, 39 and 51 [10]. The distribution of HPV genotypes was different in different regions and countries, which had led to geographic changes in the incidence and mortality of cervical cancer [11].

Primary prevention (preventive HPV vaccination) and secondary prevention (combined with effective HPV testing and screening to treat cervical precancerous lesions) are very effective [1]. At present, in resource-rich countries, the incidence of cervical cancer didn't continue to increase after the age of 40, which may well indicate that cancer can be prevented through screening [1]. As one of the largest developing countries in the world, China needs to make further advances in the prevention and screening of cervical cancer. Therefore, this study analyzed the epidemiological characteristics and long-term trends of HPV infection subtypes in Zhoupu District, Shanghai, especially for patients with abnormal cytological examination results. Analyzing the relationship between HPV genotypes and cytological examination results will help the data of the specific prevalence of HPV genotypes in Shanghai to encourage the implementation of HPV vaccine programs.

## Methods

### Data source

We collected the cervical exfoliations data of 23,724 women who visited Shanghai Zhoupu hospital and received both ThinPrep cytology test (TCT) and HPV detection between 2014 and 2019.

## Ethics statement

The study was approved by the Institutional Medical Ethics Review Committee of Shanghai Zhoupu Hospital. All 23,724 patients have signed informed consent. For patients younger than 18 years, their parents have signed a consent form. Confidentiality was ensured during the data collection process and the data were analyzed anonymously.

### Cytology testing

During non-menstrual periods, samples of cervical exfoliation were collected. Experienced Cytology experts in gynecology conducted cytology based on cervical fluid.

According to the definition by the Bethesda system, liquid-based cytological terminality includes intraepithelial lesions or malignant tumors (NILM), Low-grade squamous intraepithelial lesion (LSIL) and high-squamous intraepithelial lesion (HSIL), atypical squamous cells of undetermined significance (ASC-US), atypical squamous cells (ASC-H) which HSIL cannot be excluded, atypical glandular cells (AGC), and adenocarcinoma.

### HPV genotyping

HPV typing test kits (Certificate No. of China Food and Drug Administration (2014): 3,402,188), using PCR membrane hybridization, perform HPV genotyping on collected samples.

PCR membrane hybridization can detect 21 HPV genotypes (15 h: 16, 18, 31, 33, 35, 39, 45, 51, 52, 56, 58, 59, 66, 68 and 6 LR: 6, 11, 42, 43 44 and 81), through reverse dot hybridization and envelope specific probe membrane for hybridization.

### Statistical analysis

All HPV and TCT data from 2014 to 2019 were combined into an Excel spreadsheet and then analyzed and plotted on the R platform (www.r-project.org) (v3.2.0) and R packages.

HPV positive infection rate, single-type HPV infection rate and multi-type HPV infection rate were compared in three ways: stratified by age group (< 30 years old, 30–40 years old, 40–50 years old, 50–60 years old, ≥ 60 Years) comparison, by every HPV subtype infection, and by long-term infection trend of all 21 HPV subtypes from 2014 to 2019. At the same time, the positive rate of TCT infection in different age groups was compared according to the same age stratification.

The secular trends for TCT and HPV positive infection rates and their distribution in different age groups were both analyzed during 2014–2019. Comparisons between TCT test results and HPV subtype infections were calculated, too. In the secular trend calculation, student t-test was used and *p* < 0.05 was considered statistically significant.

## Results

### Characteristics of the study participants

In this study, a total of 23,724 women from Shanghai Zhoupu Hospital underwent outpatient gynecological examinations and were tested for HPV and TCT. Participants were between 15 and 94 years old, with an average age of 36.76.

Among the 23,724 participants, 3816 were HPV infection positive, the total HPV infection rate was 16.08% (3816/23,724), and the HR-HPV positive rate was 14.18% (3363)/23,724). Therefore, 88.13% of HPV infections are caused by HR-HPV.

Among all 23,724 participants, 18.98% (4502/23,724) women were TCT positive, of whom, 17.71% (4202/23,724), 0.06% (15/23,724), 0.53% (125/23,724), 0.67% (159/23,724), and 0.004% (1/23,724) had ASCs, AGCs, HSILs, LSILs and, Cervical adenocarcinoma, respectively.

**Fig. 1:**
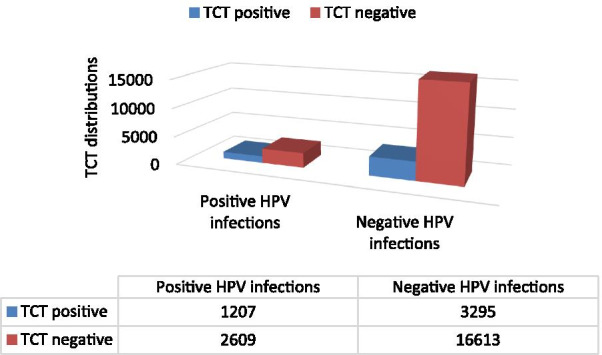
TCT cytology test results’ distributions between positive and negative HPV infection rates. The left group represents TCT distribution in HPV- positive infections, and the right group represents TCT distribution in HPV- negative infections. Dark blue indicates the number of TCT-positive infections (including AGC, ASC, LSIL, HILM) in this group, and dark red indicates the number of TCT-negative infections (NILM)

Figure 1 compares the distribution of TCT cytology results between HPV positive and negative infection rates. Among 3816 positive HPV infections, there were 31.63% (1207/3816) TCT positive rate and 68.37% (2609/3816) TCT negative. Whereas, among 19,908 negative HPV infections, there were 16.55% (3295/19,908) TCT positive infection and 83.45% (3295/19,908) TCT negative among 19,908 negative HPV infections. There was a significant difference in TCT results between HPV positive group and HPV negative group (*p* < 0.001). The positive rate of TCT in HPV-positive patients (31.63%) is much higher than that in HPV-negative patients (16.55%).

### Single-type and multiple-type HPV infections in women of different ages

Distribution of HPV single-type and multiple-type infection in different age groups are shown in Table 1. Among 2513 women aged 50–60 years, the HPV positive rate was 19.18% (482 / 2513), which was higher than that in other age groups.

**Table 1:** Distribution of HPV single-type and multiple-type infection in different age groups

Age (years)	N	HPV Positive, n (%)	Single-type infection, n (%)	Multiple-type infection, n (%)
< 30	7568	1271 (16.79)	1163 (15.37)	108 (1.43)
30–40	7866	1127 (14.33)	1043 (13.26)	84 (1.07)
40–50	4689	741 (15.8)	690 (14.72)	51 (1.09)
50–60	2513	482 (19.18)	418 (16.63)	64 (2.55)
> 60	1088	195 (17.92)	166 (15.26)	29 (2.67)
Total	23,724	3816	3480	336

Single-type infection is much higher than multiple-type infection, which are 91.20% (3480/3816) and 8.8% (336/3816) respectively (χ2 test *p* < 0.0001). Among single-type HPV infections, it is more common in women aged 50–60 and women under 30, which are 16.63% (418/2513) and 15.37% (1163/7568), respectively. Among multi-type HPV infections, women aged over 60 and 50–60 are more common, with 2.67% (29/10,88) and 2.55% (64/2513) respectively.

### Single-type and multiple-type infection rates in every subtype of all 21 HPV subtypes

**Fig. 2:**
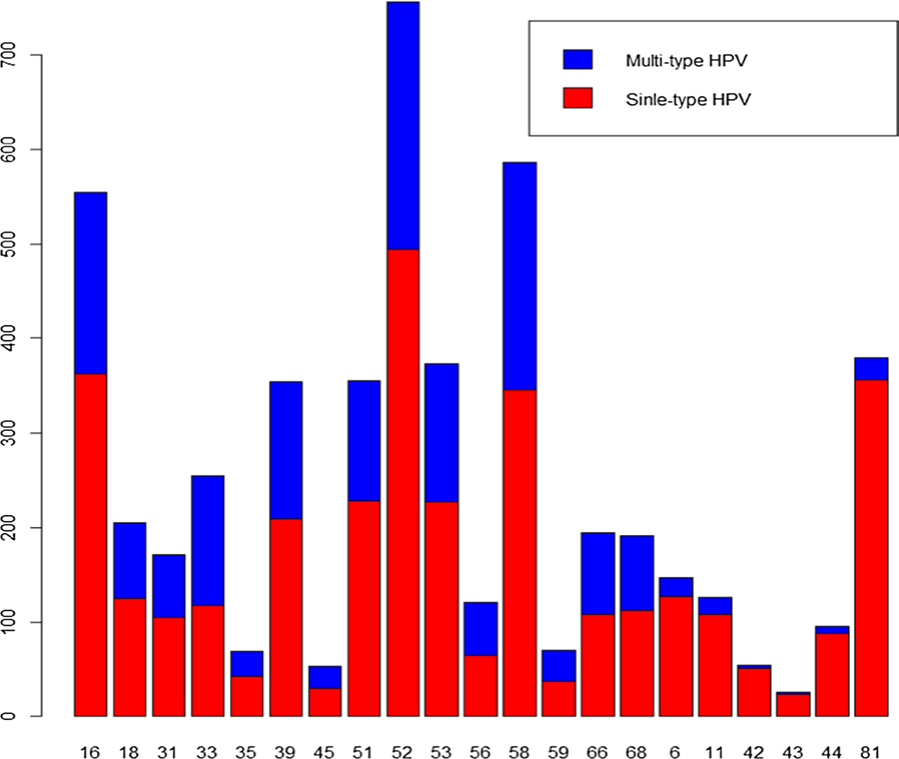
Distribution of single-type and multiple-type infection of 21 HPV subtypes. Every bar represents the number of infections of every HPV subtype. The red in each bar indicates the number of single-type infections for this HPV subtype, and the blue indicates that of the multiple-type infections

Table 2 and Fig. 2 show the comparison of the distribution of single-type and multiple-type infection rate of each subtype among all 21 HPV subtypes, and the first 15 was HR-HPV subtypes and the last 6 was LR-HPV ones. Here, the infection rate of each HPV subtype is calculated by calculating the ratio of the number of people infected by the HPV subtype to the total number of women (N = 23,724).

**Table 2:** Distribution of single-type and multiple-type infection rate of each subtype among all 21 HPV subtypes

HPV subtype	Positive n (%)	Single-type infection n (%)	Multi-type infection n (%)
HPV16	555 (2.34)	363 (1.53)	192 (0.81)
HPV18	205(0.86)	125 (0.53)	80 (0.34)
HPV31	171 (0.72)	S105 (0.44)	66 (0.28)
HPV33	255 (1.07)	118 (0.5)	137 (0.58)
HPV35	69 (0.29)	43 (0.18)	26 (0.11)
HPV39	354 (1.49)	209 (0.88)	145 (0.61)
HPV45	53 (0.22)	30 (0.13)	23 (0.1)
HPV51	355 (1.5)	228 (0.96)	127 (0.54)
HPV52	756 (3.19)	494 (2.08)	262 (1.1)
HPV53	373 (1.57)	227 (0.96)	146 (0.62)
HPV56	121 (0.51)	65 (0.27)	56 (0.24)
HPV58	586 (2.47)	346 (1.46)	240 (1.01)
HPV59	70 (0.3)	37 (0.16)	33 (0.14)
HPV66	195 (0.82)	108 (0.46)	87 (0.37)
HPV68	192 (0.81)	112 (0.47)	80 (0.34)
HPV6	147 (0.62)	12 7 (0.54)	20 (0.08)
HPV11	126 (0.53)	108 (0.46)	18 (0.08)
HPV42	54 (0.23)	51 (0.21)	3 (0.01)
HPV43	26 (0.11)	24 (0.1)	2 (0.01)
HPV44	95 (0.4)	88 (0.37)	7 (0.03)
HPV81	379 (1.6)	356 (1.5)	23 (0.1)

The first 15 were HR-HPV subtypes and the last 6 were LR-HPV subtypes

Among the 21 HPV subtypes, the top three highest prevalence rates were all high-risk HPV: HPV52, 58, and 16, with infection rates of 3.19%, 2.47% and 2.34%, respectively. Among single-type and multiple-type HPV infections, these three high-risk HPVs were also the most common.

*The trend of infection rates of different HPV subtypes in 2014–2019.*

**Figure3:**
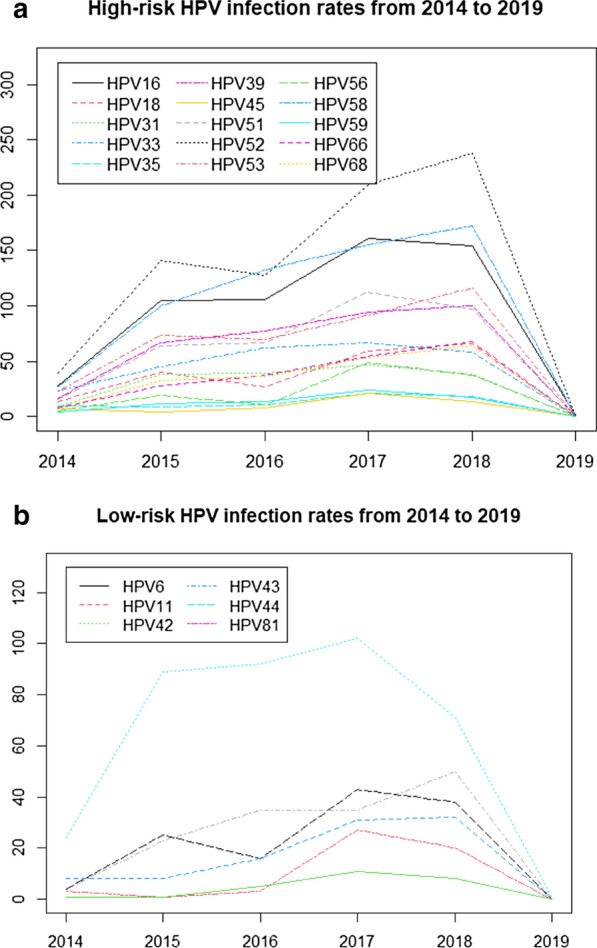
Secular trends of different HPV subtype infection rates from 2014 to 2019. **a **High-risk HPV infection rates. **b** Low-risk HPV infection rates

Table 3 and Fig. 3 show the trends of HPV subtype infections from 2014 to 2019. In the past six years, hpv52, 58, and 16 were in the top three with the highest infection rate. The trend of most subtypes infection rates had a significant difference (*p* < 0.05), except hpv42 and 43 (Table 3).

**Table 3:** Secular trends in infection rates of different HPV subtypes from 2014 to 2019

HPV subtype	2014, n (%)	2015, n (%)	2016, n (%)	2017, n (%)	2018, n (%)	2019, n (%)	*p *Value for trend
HPV16	28 (2.54)	105 (2.59)	106 (2.06)	161 (2.53)	154 (2.19)	1 (3.23)	0.0179
HPV18	13 (1.18)	40 (0.99)	27 (0.53)	59 (0.93)	66 (0.94)	0 (0)	0.0229
HPV31	9 (0.82)	38 (0.94)	39 (0.76)	47 (0.74)	38 (0.54)	0 (0)	0.0147
HPV33	23 (2.08)	45 (1.11)	62 (1.21)	67 (1.05)	58 (0.82)	0 (0)	0.0105
HPV35	10 (0.91)	9 (0.22)	11 (0.21)	21 (0.33)	18 (0.26)	0 (0)	0.0125
HPV39	16 (1.45)	67 (1.65)	77 (1.5)	94 (1.48)	100 (1.42)	0 (0)	0.0177
HPV45	7 (0.63)	4 (0.1)	8 (0.16)	21 (0.33)	13 (0.18)	0 (0)	0.0323
HPV51	15 (1.36)	64 (1.58)	67 (1.3)	112 (1.76)	97 (1.38)	0 (0)	0.0219
HPV52	39 (3.53)	141 (3.48)	128 (2.49)	209 (3.29)	238 (3.38)	1 (3.23)	0.0207
HPV53	23 (2.08)	73 (1.8)	70 (1.36)	91(1.43)	116 (1.65)	0 (0)	0.0167
HPV56	5 (0.45)	19 (0.47)	11 (0.21)	49 (0.77)	37 (0.53)	0 (0)	0.0496
HPV58	27 (2.45)	100 (2.47)	132 (2.57)	155 (2.44)	172 (2.44)	0 (0)	0.0189
HPV59	4 (0.36)	12 (0.3)	13 (0.25)	24 (0.38)	17 (0.24)	0 (0)	0.0217
HPV66	8 (0.72)	28 (0.69)	37 (0.72)	54 (0.85)	68 (0.97)	0 (0)	0.0286
HPV68	7 (0.63)	32 (0.79)	36 (0.7)	53 (0.83)	64 (0.91)	0 (0)	0.0259
HPV6	4 (0.36)	23 (0.57)	35 (0.68)	35 (0.55)	50 (0.71)	0 (0)	0.0273
HPV11	4 (0.36)	25 (0.62)	16 (0.31)	43 (0.68)	38 (0.54)	0 (0)	0.0327
HPV42	3 (0.27)	1 (0.02)	3 (0.06)	27 (0.42)	20 (0.28)	0 (0)	0.1135
HPV43	1 (0.09)	1 (0.02)	5 (0.1)	11 (0.17)	8 (0.11)	0 (0)	0.0631
HPV44	8 (0.72)	8 (0.2)	16 (0.31)	31 (0.49)	32 (0.45)	0 (0)	0.0319
HPV81	24 (2.17)	89 (2.2)	92 (1.79)	102 (1.6)	71 (1.01)	1 (3.23)	0.0132
Total	278	924	991	1466	1475	3	

The first 15 was HR-HPV subtypes and the last 6 were LR-HPV subtypes

As shown in Fig. 3, from 2014 to 2019, the prevalence of most HR-HPV was higher than that of LR-HPV. Most of the highest prevalence rate of each HPV subtype occurred in 2018. Especially HPV52 has the highest prevalence rate (3.38%) of all HPV subtypes.

### Distribution of TCT positive infections in different age groups

Comparison of the distribution of TCT positive infections in different age groups are shown in Table 4. Among women over 60 years old, the positive rate of TCT (23.05%) was the highest. And among women under 30, the positive rate of TCT (17.83%) was the lowest. Besides, the distributions of both HSIL and ASC increased significantly in different age groups (*p* < 0.0001, Table 4).

**Table 4:** Distribution of TCT positive infections in different age groups

Age (years)	N	NILM, n (%)	LSIL, n (%)	HSIL, n (%)	AGC, n (%)	ASC, n (%)	Cervical adenocarcinoma, n (%)	TCT positive, n (%)
< 30	7567	6218 (82.17)	55 (0.73)	8 (0.11)	4 (0.05)	1282 (16.94)	0 (0)	1349 (17.83)
30–40	7866	6437 (81.83)	47 (0.6)	34 (0.43)	3 (0.04)	1345 (17.1)	0 (0)	1429 (18.17)
40–50	4689	3755 (80.08)	37 (0.79)	38 (0.81)	4 (0.09)	855 (18.23)	0 (0)	934 (19.92)
50–60	2513	1974 (78.55)	17 (0.68)	30 (1.19)	3 (0.12)	489 (19.46)	0 (0)	539 (21.45)
> 60	1089	838 (76.95)	3 (0.28)	15 (1.38)	1 (0.09)	231 (21.21)	1 (0.09)	251 (23.05)
Total	23,724	19,222	159	125	15	4202	1	4502

*Distribution of HPV and TCT infection rates in different age groups from 2014 to 2019.*

**Fig. 4:**
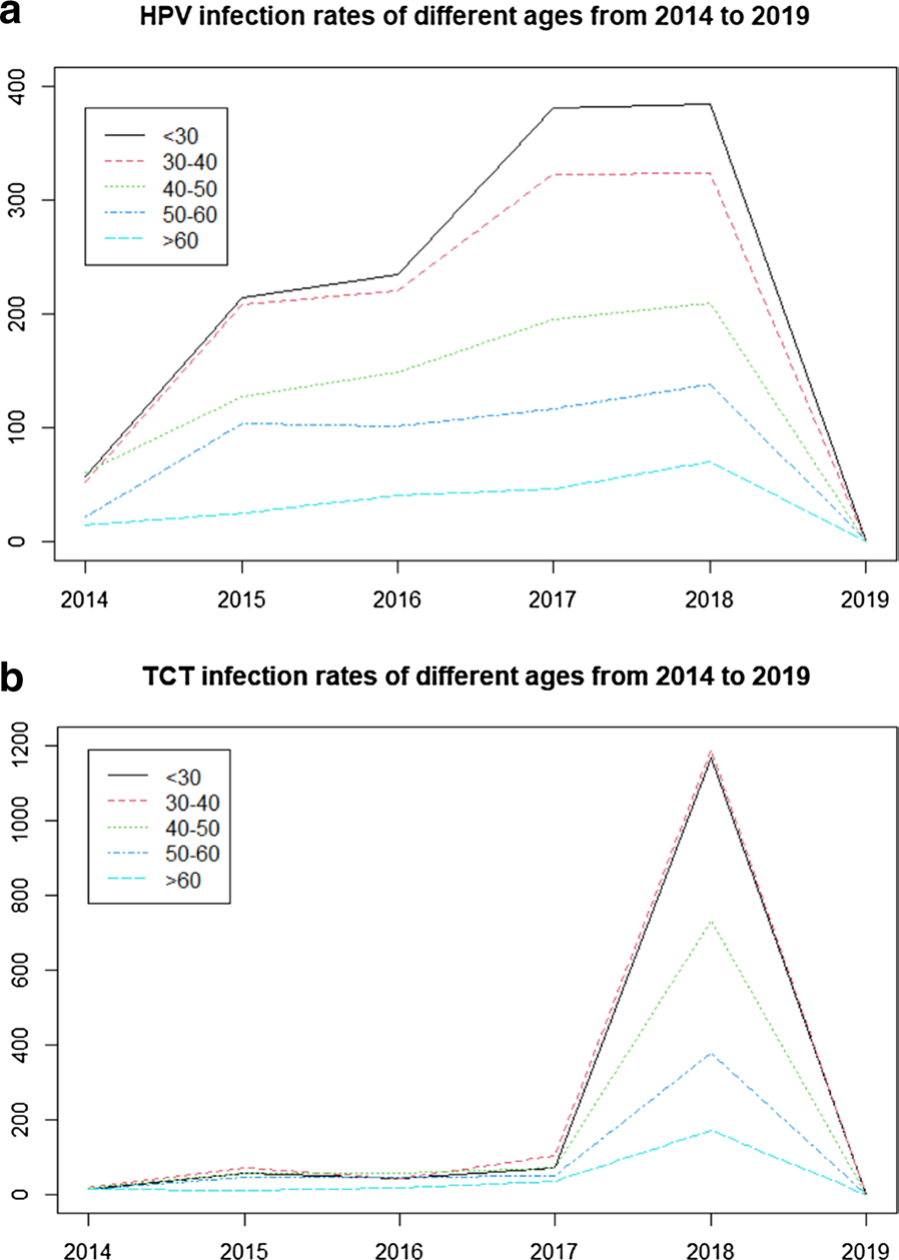
Distribution of HPV and TCT infection rates in different age groups from 2014 to 2019. **a** HPV infection distribution. **b** TCT infection distribution

Table 5 and Fig. 4 show the distribution of HPV and TCT infection rates in different age groups from 2014 to 2019.

**Table 5:** Distribution of HPV and TCT infection rates in different age groups from 2014 to 2019

Age (years)	2014, n (%)	2015, n (%)	2016, n (%)	2017, n (%)	2018, n (%)	2019, n (%)	*p* Value for trend
HPV positive							
< 30	57 (27.8)	214 (31.56)	234 (31.45)	381 (35.91)	384 (34.13)	1 (33.33)	0.0226
30–40	52 (25.37)	208 (30.68)	220 (29.57)	322 (30.35)	324 (28.8)	1 (33.33)	0.0192
40–50	61 (29.76)	127 (18.73)	149 (20.03)	195 (18.38)	209 (18.58)	0 (0)	0.0131
50–60	21 (10.24)	104 (15.34)	101 (13.58)	117 (11.03)	138 (12.27)	1 (33.33)	0.0166
> 60	14 (6.83)	25 (3.69)	40 (5.38)	46 (4.34)	70 (6.22)	0 (0)	0.0241
Total	205	678	744	1061	1125	3	-
TCT positive							
< 30	14 (17.5)	56 (23.14)	43 (20.38)	70 (21.08)	1166 (32.07)	0 (0)	0.2866
30–40	20 (25)	73 (30.17)	44 (20.85)	103 (31.02)	1188 (32.67)	1 (100)	0.2667
40–50	17 (21.25)	57 (23.55)	58 (27.49)	72 (21.69)	730 (20.08)	0 (0)	0.2353
50–60	15 (18.75)	46 (19.01)	47 (22.27)	52 (15.66)	379 (10.42)	0 (0)	0.1849
> 60	14 (17.5)	10 (4.13)	19 (9)	35 (10.54)	173 (4.76)	0 (0)	0.1773
Total	80	242	211	332	3636	1	-

We found that the HPV positive rate of all women has changed significantly from 2014 to 2019 (P-value for trend < 0.05). whereas, there was no significant difference in the TCT positive rate during this period (Table 5).

From Fig. 4, except for 2018, the number of TCT infections was lower than the number of HPV infections. In 2018, there were 384 HPV infections and 1166 TCT infections among women under 30 years of age, and 324 HPV infections and 1188 TCT infections among women 30–40 years old.

### The relationship between TCT test results and each HPV subtype infections

**Fig. 5:**
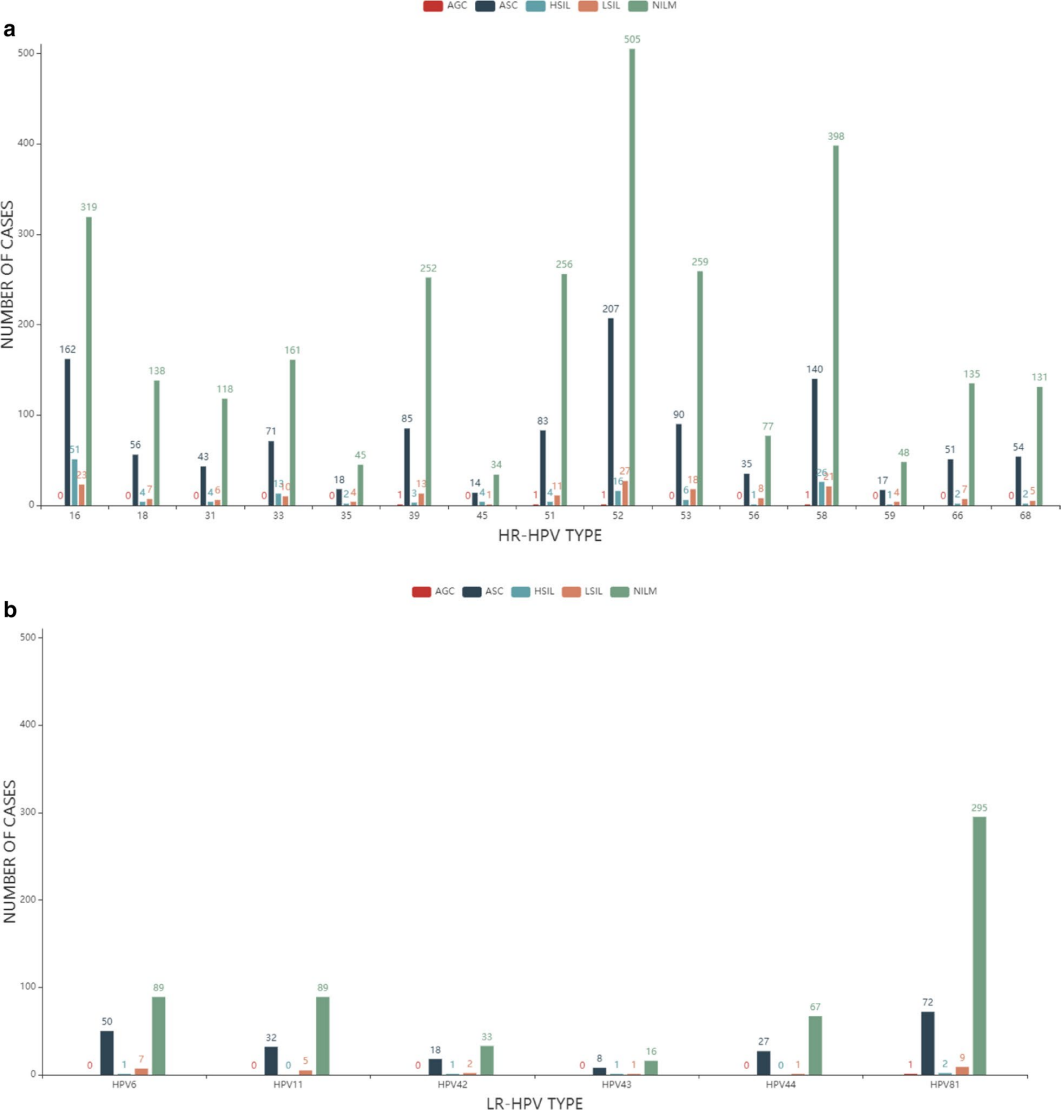
Distribution of every HPV subtype infection rates among NILM, AGC, ASC, LSIL, and HSIL. **a** Distribution of every HR-HPV infection rate for all TCT test results. **b** Distribution of every LR-HPV infection rate for all TCT test results

Figure 5 shows the distribution of every HPV subtype infection rates among NILM, AGC, ASC, LSIL, and HSIL. Figure 5a shows every HR-HPV infection number for all TCT test results, and Fig. 5b shows every LR-HPV infection. Among the four abnormal TCT results, ASC infection (Dark blue) has the highest number of infections both in HR-HPV and LR-HPV. Moreover, there are more TCT positives in HR-HPV patients than LR-HPV patients. Few HPV-positive patients have AGC infection (red).

The last column of Table 6 shows the top three HPV subtypes with the highest TCT positive rate were HPV 16, 6, and 42 (42.52%, 39.46% and 38.89%), among the patients who received both HPV and TCT examinations. This is different from the second column of Table 6. Among HPV-positive patients, the subtypes with the highest prevalence are 52, 58, and 16.

**Table 6:** Different HPV subtype infection rates among NILM, AGC, ASC, LSIL, and HSIL, respectively

HPV subtype	N	NILM (%) N = 19,222	LSIL (%) N = 159	HSIL (%) N = 125	AGC (%) N = 15	ASC (%) N = 4202	Cervical adenocarcinoma (%) N = 1	TCT positive, n (%)
HPV16	555	319 (57.48)	23 (4.14)	51 (9.19)	0 (0)	162 (29.19)	0 (0)	236 (42.52)
HPV18	205	138 (67.32)	7 (3.41)	4 (1.95)	0 (0)	56 (27.32)	0 (0)	67 (32.68)
HPV31	171	118 (69.01)	6 (3.51)	4 (2.34)	0 (0)	43 (25.15)	0 (0)	53 (30.99)
HPV33	255	161 (63.14)	10 (3.92)	13 (5.1)	0 (0)	71 (27.84)	0 (0)	94 (36.86)
HPV35	69	45 (65.22)	4 (5.8)	2 (2.9)	0 (0)	18 (26.09)	0 (0)	24 (34.78)
HPV39	354	252 (71.19)	13 (3.67)	3 (0.85)	1 (0.28)	85 (24.01)	0 (0)	102 (28.81)
HPV45	53	34 (64.15)	1 (1.89)	4 (7.55)	0 (0)	14 (26.42)	0 (0)	19 (35.85)
HPV51	355	256 (72.11)	11 (3.1)	4 (1.13)	1 (0.28)	83 (23.38)	0 (0)	99 (27.89)
HPV52	756	505 (66.8)	27 (3.57)	16 (2.12)	1 (0.13)	207 (27.38)	0 (0)	251 (33.2)
HPV53	373	259 (69.44)	18 (4.83)	6 (1.61)	0 (0)	90 (24.13)	0 (0)	114 (30.56)
HPV56	121	77 (63.64)	8 (6.61)	1 (0.83)	0 (0)	35 (28.93)	0 (0)	44 (36.36)
HPV58	586	398 (67.92)	21 (3.58)	26 (4.44)	1 (0.17)	140 (23.89)	0 (0)	188 (32.08)
HPV59	70	48 (68.57)	4 (5.71)	1 (1.43)	0 (0)	17 (24.29)	0 (0)	22 (31.43)
HPV66	195	135 (69.23)	7 (3.59)	2 (1.03)	0 (0)	51 (26.15)	0 (0)	60 (30.77)
HPV68	192	131 (68.23)	5 (2.6)	2 (1.04)	0 (0)	54 (28.12)	0 (0)	61 (31.77)
HPV6	147	89 (60.54)	7 (4.76)	1 (0.68)	0 (0)	50 (34.01)	0 (0)	58 (39.46)
HPV11	126	89 (70.63)	5 (3.97)	0 (0)	0 (0)	32 (25.4)	0 (0)	37 (29.37)
HPV42	54	33 (61.11)	2 (3.7)	1 (1.85)	0 (0)	18 (33.33)	0 (0)	21 (38.89)
HPV43	26	16 (61.54)	1 (3.85)	1 (3.85)	0 (0)	8 (30.77)	0 (0)	10 (38.46)
HPV44	95	67 (70.53)	1 (1.05)	0 (0)	0 (0)	27 (28.42)	0 (0)	28 (29.47)
HPV81	379	295 (77.84)	9 (2.37)	2 (0.53)	1 (0.26)	72 (19)	0 (0)	84 (22.16)
Total	5137	3465	190	144	5	1333	0	1672

The first 15 were HR-HPV subtypes and the last 6 were LR-HPV subtypes

In Table 6, among the five cytopathies, the HPV infection rates corresponding to NILM, LSIL, HSIL, AGC and ASC were 66.08% (3495/19,222), 119.50% (190/159), 115.20% (144/125), and 63.99%, respectively (5/15) and 31.72% (1333/4202)). And the HPV infection rates corresponding to LSIL and HSIL were both greater than 100%, which means that there are many mixed multi-subtype HPV infections in these two types of cytological lesions.

In NILM, the most common HPV-positive subtypes are 51, 39, and 16. In HSIL, the most common HPV-positive subtypes are 45,33 and 58. In LSIL, the most common HPV-positive subtypes are 56,35 and 59. In ASC, the most common HPV-positive subtypes are 6.42 and 43. Very few HPV positive were found in AGC (Table 6) (Fig. 5).

### Comparison of TCT positive infection in single-type and multiple-type HPV infection

**Fig. 6:**
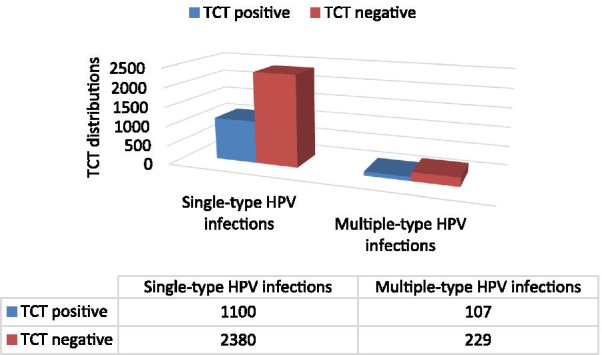
TCT positive rates distribution between single-type and multiple-type HPV infections. The left group represents TCT distribution in single-type HPV infection, and the right group represents TCT distribution in multiple-type HPV infection. Dark blue indicates the number of TCT-positive infections (including AGC, ASC, LSIL, HILM) in this group, and dark red indicates the number of TCT-negative infections (NILM)

In Fig. 6, according to the classification of HPV infection in single-type and multiple-type, the positive rates of TCT were 31.61% (1100/3480) and 31.84% (107/336), respectively. The proportions of TCT positive between single-type and multiple-type are almost the same.

## Discussion

This study analyzed the HPV epidemic characteristics of 23,724 women who underwent TCT and HPV testing in Zhoupu District, Shanghai from 2014 to 2019. The overall positive rate of HPV, the positive rate of single-type HPV infection and multiple-type infection were 16.08%, 91.2% (3480/3816) and 8.8% (336/3816), respectively.

Our research showed that the infection rate of single-type HPV peaks in women aged 50–60 years, and ranks second in women under 30 years old, and the infection rate of multiple-type HPV peaks in women over 60 years old, and ranks second in women 50–60 years old. HR- HPV52, 58 and 16 were the most common subtypes both in the single-type and multiple-type HPV infections.

Our research showed that the women’s overall prevalence of HPV was 16.08%, lower than 18.98% in Songjiang District [5, 6]. However, the HPV infection rate in the Minhang District is higher (73.5%) [4], maybe because there are fewer patients involved (147 in the Minhang District, 23,724 in our study, 33,562 in the Songjiang District).

Our results were different from Xinjiang [12], Guizhou [13], Beijing [14] and Suzhou [15]. Xinjiang and Guizhou are both less-developed provinces in China where simple HPV infection and multiple-type were both common in younger women [12, 13]. In Beijing, single-type HPV infection was more frequently seen in women aged 50–60 years, and multiple-type HPV infection was more common in those aged < 30 years, but the HR-HPV subtypes were similar to ours [14]. In Suzhou [15], HR-HPV infection rate was a peak in women aged < 20 years, and second higher in women aged 51–60 years, infection modes were HPV16, 18, 31, 33, 45, 52, 58. One reason for the difference could be the peak or plateau in cervical cancer incidence from ages 35 to 55 years [16], another could be aggravating trend of the ageing population in Shanghai.

We found less HPV positive prevalence, but there was a higher overall prevalence of TCT, 18.98%. The TCT positive rate of ASCs, AGCs, HSILs, LSILs and Cervical adenocarcinoma were 17.71%, 4.04%, 2.64%,1.54%, and 0.004%, respectively. The highest positive rate of TCT is 23.05% in women aged ≥ 60 years, and the lowest positive rate was 17.83% in those aged <  = 30 years. Moreover, TCT positive ratio was 16.55% (3295/19,908) even in the negative HPV infection women. The data from Beijing showed that the TCT positive ratio was 4.04%,2.64%,1.54% for ASC, LSIL, HSIL, respectively. The data from Mongolia [17], a developing country in Asia, presented that HR-HPV 16, 52, 58, and 33 were common. Cytological examination results revealed that the positive rate was 12%, 8% 7%,14% for ASC, LSIL, HSIL, and squamous cell carcinoma. Moreover, in our study, the TCT positive rates of the single-type and multiple-type HPV infections had no difference (31.61% vs 31.84%), which suggests that the increase in the number of HPV subtype infections does not increase the risk of TCT positive infection.

Similar studies have previously examined HPV prevalence in women in Shanghai areas. Our research showed that the HPV detection rate between 2014 and 2019 was 66.08% (3495/19,222) in normal cervical tissue, 119.50% (190/159) in the LSIL group, 115.20% (144/125) in the HSIL group. In the Minhang district, the HPV infection rate between January and December 2016 was 73.5%(108/147), of which the HPV detection rate was 5.0%(1/20)in normal cervical tissue, 67.3%(35/52) in the LSIL group, 95.7%(44/46)in the HSIL group [4]. Consistent with the Minhang district, the HPV infection rate also gradually increased according to the grade of cervical neoplasia. In particular, the HPV infection rate in the LSIL and HSIL groups in the two districts was very high (> 95%), and the infection rate in our study is even greater than 100% (more multi-subtype HPV mixed infection occurred).

In our study, including 23,724 women, HPV 45, 33 and 58 were frequently determined in HSIL; HPV 56, 35 and 59 were commonly found in LSIL; HPV 51, 39 and16 were frequently determined in NILM; which was consistent with the several studies [18–21]. In the Minhang District of Shanghai, HR-HPV (16, 18, 31, 33, 52, and 58) were only identified in HSIL; 12 h-HPV genotypes(16, 18, 31, 33, 35, 39, 45, 51, 52, 53, 56, 58, 59, 66,68, 73, and 82) were detected in cases with LSIL; only 1 HPV6 infection was identified in NILM [4]. One study included 3143 cases from Beijing showed that HPV subtype 16, 58, 52, 31 and 51 were commonly found in HSIL; HPV16, 52, 58, 56 and 51 in LSIL; HPV subtype 16, 31, 6,11, 52 and 58 in NILM. Another study involved 18,170 women from Korea reported that HPV 16 was common types in HSIL; HPV 58,53,56,51 in LSIL; HPV 58,52,53,16 in ASC. The natural history model of Cervical Cancer has shown that the relative significance of HPV16 increased with lesion severity, especially in HSIL [16]. The low-risk HPV genotypes 6 and 11 induce genital warts or condylomas, HPV 6 also were commonly found in ASC in our study. Therefore, HPV and TCT screening is the key step of secondary prevention of cervical cancer. Tracking the results of these two screening is an important clinical strategy for the treatment of cervical precancerous lesions.

First-generation vaccines directly protect against oncogenic HPV types 16 and 18 in individuals naive for those types, and these two HPV subtypes are responsible for approximately 70% of invasive cervical cancer.[22]. Recently, it has been shown that second-generation 9-valent vaccine against the HPV types 6,11,16,18,31,33,45,52,58 which were responsible for up to 90% of cervical cancers can either directly protect against a larger proportion of types [23, 24]. However, in 2016, only 14% of low-income and low-middle-income countries had vaccination programs [25]. Only three years ago, China obtained the first batch of HPV vaccine license, and China has not yet decided whether to include the HPV vaccine in its routine immunization program [26]. From our study, the top three with the highest prevalence were HPV52, 58 and 16. Most CIN 2 + lesions are caused by HR-HPV gene subtypes, especially those covered by the 9-valent vaccine. Therefore, the widespread use of the HPV vaccine can greatly reduce the incidence of cervical precancerous lesions and neoplastic lesions.

Our study also has some limitations. First, although HPV gene subtypes are biologically independent, some of these subtypes may have common biological characteristics, resulting in similar phenotypes (such as the similarity of the number of infected people). Therefore, further analysis should be made to investigate the similarity of infection for HPV sub-types infection in the cytology test results. Second, although the relationship between HPV infection and TCT cytology results is the focus of this study, the characteristics of bacterial vaginosis infection were not involved. So, the association among HPV infection, abnormal cervical cytology and bacterial vaginosis should be considered.

## Conclusions

In descending order, the three most common HR-HPVs are HPV52, 58, and 16. Single-type infection was more common than multitype infection. The long-term trend analysis of data from 2014 to 2019 showed that the HPV positive rate had changed significantly. HPV and TCT screening were very important steps in the secondary prevention of cervical cancer. Through the tracking and analysis of HPV and TCT results in this study, it can provide valuable information for Shanghai's HPV screening and prevention strategies, and provide references for clinical decision-making in the treatment of cervical cancer and precancerous lesions. The results of our research also indicate that there is still a need to further promote the wide application of preventive HPV vaccines, which will greatly help to significantly reduce the occurrence of precancerous lesions and neoplastic cervical lesions.

## Data Availability

The data was collected from Shanghai Zhoupu Hospital. We thank them for their generous help. Data can be shared for free. The materials were purchased from Hybribio Biotechnology Limited Corp and LBP Medicine Science & Technology Co., Ltd.

## Abbreviations

HPV: Human papillomavirus
HR-HPV: High-risk HPV
NILM: Intraepithelial lesions or malignancy
AGCs: Atypical glandular cells
ASC-US: Atypical squamous cells of undetermined significance
LSIL: Low-grade squamous intraepithelial lesions
HSIL: High-grade squamous intraepithelial lesions
TCT: ThinPrep cytological test

## References

[1] Arbyn M, Weiderpass E, Bruni L, de Sanjosé S, Saraiya M, Ferlay J, Bray F (2020). Estimates of incidence and mortality of cervical cancer in 2018: a worldwide analysis. Lancet Glob Health.

[2] Wei M, Zhou W, Bi Y, Wang H, Liu Y, Zhang ZJ. Rising mortality rate of cervical cancer in younger women in urban China. J Gen Internal Med, 2019; 34(2), 281-284 10.1007/s11606-018-4732-z PMC6374275 30484099

[3] Chen W, Zheng R, Baade PD, Zhang S, Zeng H, Bray F, He J. Cancer statistics in China15. CA, 2016; 66(2): 115-132. 10.3322/caac.21338 26808342

[4] Wu J, Li X, Liu X, Gao Z (2018). Human papillomavirus genotype prevalence in the women of Shanghai, China and its association with the severity of cervical neoplasia. Int J Clin Exp Pathol.

[5] Zhang C, Zhang C, Huang J, Wu Z, Mei X, Shi W (2018). Prevalence and genotype distribution of human papillomavirus among females in the suburb of Shanghai China. J Med Virol.

[6] Zhang C, Zhang C, Huang J, Shi W (2018). The genotype of human papillomavirus and associated factors among high risk males in Shanghai, China: a molecular epidemiology study. Med Sci Monitor.

[7] Li X, Deng Y, Tang W, Sun Q, Chen Y, Yang C, Cao G. Urban-rural disparity in cancer incidence, mortality, and survivals in Shanghai, China, during 2002 and 2015. Front Oncol, 2018;8, 579 10.3389/fonc.2018.00579 PMC6287035 30560091

[8] Zhao FH, Lewkowitz AK, Hu SY, Chen F, Li LY, Zhang QM, Franceschi S. Prevalence of human papillomavirus and cervical intraepithelial neoplasia in China: a pooled analysis of 17 population-based studies. Int J Cancer 2012, **131**: 2929–2938. 10.1002/ijc.27571 PMC3435460 22488743

[9] Giorgi Rossi P, Baldacchini F, Ronco G. The possible effects on socio-economic inequalities of introducing HPV testing as primary test in cervical cancer screening programs. Front Oncol 2014,4:20. 10.3389/fonc.2014.00020 PMC3919018 24575388

[10] Li H, Li P, Huang L, Sun L, Ren H, Li P. Prevalence characteristics of cervical human papillomavirus (HPV) infection in the Zhoupu District, Shanghai City, China. Virol J 2020, 17:84. 10.1186/s12985-020-01352-8 PMC7318542 32586352

[11] Bruni L, Diaz M, Castellsagué X, Ferrer E, Bosch F, de Sanjosé S (2010). Cervical human papillomavirus prevalence in 5 continents: meta-analysis of 1 million women with normal cytological findings. J Infect Dis.

[12] Wang J, Tang D, Wang K, Wang J, Zhang Z, Chen Y, Ma C. HPV genotype prevalence and distribution during 2009–2018 in Xinjiang, China: baseline surveys prior to mass HPV vaccination. BMC Women's Health 2019, 19:90. 10.1186/s12905-019-0785-3 PMC6615222 31286939

[13] Wang J, Tang D, Wang K, Wang J, Zhang Z, Chen Y (2019). HPV genotype prevalence and distribution during 2009–2018 in Xinjiang, China: baseline surveys prior to mass HPV vaccination. BMC Women's Health.

[14] Ma L, Lei J, Ma L, Cong X, Wang N, Yang H, Cao Y. Characteristics of women infected with human papillomavirus in a tertiary hospital in Beijing China, 2014–2018. BMC Infect Dis 2019, 19:670. 10.1186/s12879-019-4313-8 PMC6664751 31357941

[15] Wj L, Hx X, Zh C, Wd X, Yj W (2017). Characteristics of carcinogenic human papillomavirus infection in Suzhou: Epidemiology, vaccine evaluation, and associated diseases. J Med Virol.

[16] Campos NG, Burger EA, Sy S, Sharma M, Schiffman M, Rodriguez AC, Kim JJ. An updated natural history model of cervical cancer: derivation of model parameters. Am J Epidemiol 2014, 180: 545–555. 10.1093/aje/kwu159 PMC4143081 25081182

[17] Tsedenbal B, Yoshida T, Enkhbat B, Gotov U, Sharkhuu E, Saio M (2018). Human papillomavirus genotyping among women with cervical abnormalities in Ulaanbaatar, Mongolia. Int J Infect Dis.

[18] Liu Y, Ang Q, Wu H, Xu J, Chen D, Zhao H (2020). Prevalence of human papillomavirus genotypes and precancerous cervical lesions in a screening population in Beijing, China: analysis of results from China's top 3 hospital, 2009–2019. Virol J.

[19] Ouh Y, Min K, Cho H, Ki M, Oh J, Shin S (2018). Prevalence of human papillomavirus genotypes and precancerous cervical lesions in a screening population in the Republic of Korea, 2014–2016. J Gynecol Oncol.

[20] Zhao X, Hu S, Zhang Q, Dong L, Feng R, Han R (2017). High-risk human papillomavirus genotype distribution and attribution to cervical cancer and precancerous lesions in a rural Chinese population. J Gynecol Oncol.

[21] Iacobone A, Bottari F, Radice D, Preti E, Franchi D, Vidal Urbinati A (2019). Distribution of high-risk human papillomavirus genotypes and multiple infections in preneoplastic and neoplastic cervical lesions of unvaccinated women: a cross-sectional study. J Lower Genital Tract Dis.

[22] Paavonen J, Naud P, Salmerón J, Wheeler C, Chow S, Apter D (2009). Efficacy of human papillomavirus (HPV)-16/18 AS04-adjuvanted vaccine against cervical infection and precancer caused by oncogenic HPV types (PATRICIA): final analysis of a double-blind, randomised study in young women. Lancet (Lond Engl).

[23] Joura E, Giuliano A, Iversen O, Bouchard C, Mao C, Mehlsen J (2015). A 9-valent HPV vaccine against infection and intraepithelial neoplasia in women. N Engl J Med.

[24] Kavanagh K, Pollock K, Cuschieri K, Palmer T, Cameron R, Watt C (2017). Changes in the prevalence of human papillomavirus following a national bivalent human papillomavirus vaccination programme in Scotland: a 7-year cross-sectional study. Lancet Infect Dis.

[25] Gallagher K, LaMontagne D, Watson-Jones D (2018). Status of HPV vaccine introduction and barriers to country uptake. Vaccine.

[26] Wei L, Su Y, Hu Y, Li R, Chen W, Pan Q*, et al.* Age distribution of human papillomavirus infection and neutralizing antibodies in healthy Chinese women aged 18–45 years enrolled in a clinical trial. Clin Microbiol Infect 2020. 10.1016/j.cmi.2019.12.010 31904566

